# Special Issue “Drug Discovery of Antiprotozoal Agents”

**DOI:** 10.3390/ph17020176

**Published:** 2024-01-30

**Authors:** Francisco Jaime Bezerra Mendonça-Junior

**Affiliations:** Laboratory of Synthesis and Drug Delivery, Paraíba State University, João Pessoa 58071-160, Brazil; franciscojaime@servidor.uepb.edu.br

Protozoal diseases, such as leishmaniasis, malaria, African sleeping sickness, Chagas disease, amoebiasis, giardiasis, cryptococcosis, and toxoplasmosis (among others), affect and/or have the potential to infect more than one billion people worldwide. Due to the fact that they present high mortality and morbidity rates, treatment options are limited, old, and not completely efficient due to the emergence of resistant strains, and with important adverse effects, they continue to be serious public health problems, especially in low-income countries in which health and sanitation conditions are precarious [1,2,3].

This scenario has created many opportunities for researchers to seek new therapeutic alternatives (new drugs) to treat these diseases. In this Special Issue, eleven articles, nine research papers, and two review articles with different approaches present alternatives for drug design and the discovery of antiprotozoan drugs.

Suárez-Rico and their research team published findings that support that terfenadine (TFN) may be a repositioned drug for the treatment of giardiasis. To reach this conclusion, the authors evaluated the cytotoxic effect of TFN on *Giardia lamblia* trophozoites and observed that TFN is capable of inhibiting the growth and cell viability of *G. lamblia* trophozoites in a time–dose-dependent manner, in addition to presenting low toxicity for Caco-2 cells. They identified an increased number of protrusions on membranes shortening flagella, and tubulin dysregulation using SEM (scanning electron microscopy). RT-PCR (real-time polymerase chain reaction) also found that TFN promotes the dysregulation of Giardia *Gi*K, which is a potential target of TFN in *G. lamblia* (contribution 1).

In a study by Arce-Fonseca et al., the researchers evaluated the possibility of repositioning the drug nitazoxanide (NTZ) in vivo for use in Chagas disease against the Mexican *Trypanossoma cruzi* Ninoa strain in BALB/c mice. The authors observed that both NTZ and the reference drug benznidazole (BNZ) were able to increase lifespan and decrease parasitemia levels in the treated animal groups. However, the NTZ-treated mice group showed lower levels of histological damage when compared to the BNZ-treated mice group. This effect may be due to the fact that NTZ promotes the production of IgG1-type antibodies, while BNZ produces IgG2-type antibodies. Therefore, NTZ showed good signs that it could be used as an alternative drug for the treatment of Chagas disease (contribution 2).

El-Wakil et al. evaluated the anticryptosporidiosis and anticancer properties in silico and in vivo of *Annona muricata* leaf herbal medicine compared to the reference drug nitazoxanide (NTZ). It was observed in the docking analysis that the secondary metabolites *L*-epigallocatechin, *P*-coumaric acid, and ellagic acid obtained lower binding free energy values (−7.51, −7.81, and −9.64 kcal/mol, respectively) than NTZ (−7.03 kcal/mol) compared to lactate dehydrogenase enzyme from *Cryptosporidium parvum*. In an infection model in vivo carried out with immunosuppressed albino mice, it was observed that *A. muricata* leaves were more effective than NTZ in reducing the number of *C. parvum* oocysts, in addition to resulting in restoring the normal villous pattern without evidence of dysplasia (contribution 3).

Monzote et al. evaluated the antileishmanial activity of essential oil from *Pimenta dioica* (EO-Pd). The authors identified 45 components in EO-Pd by GC-MS (gas chromatography coupled to a mass spectrometer), with the monoterpene eugenol being the most abundant (80.1%). The EO-Pd inhibited the growth of promastigote and amastigote forms of *L. amazonensis* in vitro with IC_50_ values of 9.7 ± 0.7 and 11.3 ± 2.1 μg/mL, respectively, and a selectivity index equal to nine. In an in vivo model of cutaneous leishmaniasis in BALB/c mice, EO-Pd and eugenol promoted infection control when administrated via the intralesional route, with eugenol being more effective than the reference drug Pentamidine^®^ (contribution 4).

Alanazi and Alnomasy prepared copper nanoparticles (CNPs) using aqueous extract of *Lupinus arcticus* in order to evaluate their effects in vitro and in vivo against chronic *Toxoplasma gondii* infection. CNP were fully characterized using physical and spectroscopic methods. CNP in vitro presented an IC_50_ value of 37.2 µg/mL against tachyzoites of *T. gondii* and a selectivity index greater than 13. The in vivo toxoplasmosis model in BALB/c mice CNP associated with pyrimethamine was capable of reducing the number and size of the *T. gondii* cysts in the infected mice and revealed no toxicity at the evaluated doses. Additionally, the anti-*T. gondii* activity of CNP was associated in vitro and in vivo with the control of oxidative stress, and by increasing the production of immunomodulatory cytokines (IFN-γ, IL-12, and iNO) (contribution 5).

Velásquez-Torres and colleagues demonstrated the in silico and in vitro anti-*Entamoeba histolytica* activities of riluzole (RLZ), a benzothiazole derivative. Through molecular docking in silico, it was observed that RLZ had high affinity for enzymes related to the prevention, regulation, and repair of oxidative damage, especially the thioredoxin (*Eh*Trx) and thioredoxin reductase (*Eh*TrxR) enzymes from *E. histolytica*. RLZ in vitro presented an IC_50_ value of 319.5 μM against *E. histolytica* trophozoites; decreased amoeba viability by 48.1%; promoted important ultrastructural changes and similar cell death to apoptosis; in addition to increasing the production of reactive oxygen species (ROS), while promoting negative gene expression regulation of amoebic antioxidant enzymes. Therefore, it is shown that RLZ may be a new drug candidate with antiamoebic activity (contribution 6).

With the aim of identifying new compounds with promising antileishmanial activity, Do Carmo Maquiaveli et al. synthesized a series of anthranyl phenylhydrazide derivatives. Thus, three of the synthesized compounds (bromine substituted) showed good activity against promastigotes forms of *L. amazonensis*, showing IC_50_ values between 1 and 5 μM. However, all were inactive against amastigotes, and were also unable to inhibit the *L. amazonensis* arginase enzyme (ARG-L). A target-fishing approach was carried out and suggested the pteridine reductase 1 (PTR1) as a possible target, which needs to be validated in further studies (contribution 7).

Our group (contribution 8) synthesized and evaluated the antileishmanial activity of the acridine derivative ACW-02. In vitro studies against *L. amazonensis* showed IC_50_ values of 6.57 and 94.97 µg mL^−1^ against amastigotes and promastigotes, respectively, and low cytotoxicity to macrophages (CC50 > 256.00 µg mL^−1^). The antileishmanial activity of ACW-02 can also be associated with slight immunomodulatory activity, since the compound altered TNF-α, IL-10, and IL-6 expression. In vitro (*ct*DNA interaction evaluation by UV–vis spectroscopy) and in silico (molecular docking and molecular dynamics simulations) studies against DNA demonstrate that DNA is a potential target of this acridine. It was observed that ACW-02 showed an interaction with the DNA minor grooves. These results were validated by in silico studies.

Finally, the group coordinated by Professor Rivera published three papers, one experimental and two reviews. The first of them is a ligand-based virtual screening (LBVS) study carried out with a group of benzimidazole derivatives extracted from the ZINC15 database. The aim of this work was to predict potential ligands facing the dimer interface of the triose phosphate isomerase of *L. mexicana* (*Lm*TIM). A total of 175 compounds were initially selected through molecular docking and molecular dynamics simulation studies. Five compounds were selected after the in silico determination of the physicochemical and pharmacokinetic properties, and two of them were evaluated in vitro against promastigotes forms of *L. mexicana*. The benzimidazole derivative “E2” with an IC_50_ value of 4.04 µM was the best of them, confirming the effectiveness of the LBVS in screening potential antileishmanial drug candidates (contribution 9).

The first review paper addresses the *Giardia lamblia* nucleolus as a target for research into new giardicidal agents. The authors located several therapeutic targets, including proteins and ncRNAs such as fibrillarin, Krr1, snoRNP, GdSir2.4, G/TBP, elF4E1, and snoRNA, which could be used as targets for the design of new specific drugs. Finally, the authors cite four molecules under research which could be effective for the treatment of giardiasis. Actinomycin D and cisplatin are potential candidates for drug repositioning, while Auranofin and 20-hydroxyecdysone are, respectively, in phases 2 and 3 of clinical studies (contribution 10).

In the last review article of this collection, Rivera’s team (contribution 11) presented and analyzed the epigenetic mechanisms that can be used as therapeutic targets to obtain new antiprotozoal drugs against seven protozoa: *Trypanosoma cruzi*, *T. brucei*, *Leishmania* spp., *E. histolytica*, *G. lamblia*, *T. gondii*, and *Trichomonas vaginalis*. In addition, the authors present recent advances in the drug discovery and development of inhibitors against each of these parasites.

In conclusion, several updates were presented, including drug repurposing, the bioprospection of natural products, in addition to the conventional synthesis and drug design strategies of medicinal chemistry, especially through rational drug design based on the ligand (LBDD), in which molecular docking and molecular dynamics play fundamental roles. Several promising compounds, plants, and biological targets were presented and will constitute an important basis for continued research in the search for new drug candidates for protozoan diseases.
